# Short- and long-term recurrence of early-stage invasive ductal carcinoma in middle-aged and old women with different treatments

**DOI:** 10.1038/s41598-022-08328-4

**Published:** 2022-03-15

**Authors:** Yuan Kao, Ying-Jhen Wu, Chien-Chin Hsu, Hung-Jung Lin, Jhi-Joung Wang, Yu-Feng Tian, Shih-Feng Weng, Chien-Cheng Huang

**Affiliations:** 1 Department of Emergency Medicine, Chi Mei Medical Center, 901 Zhonghua Road, Yongkang District, Tainan City, 710 Taiwan; 2 Department of Medicine Science Industries, Chang Jung Christian University, Tainan, Taiwan; 3 Teaching and Research Center of Kaohsiung Municipal Siaogang Hospital, Kaohsiung City, Taiwan; 4 Department of Emergency Medicine, Taipei Medical University, Taipei, Taiwan; 5 Department of Anesthesiology, Chi Mei Medical Center, Tainan, Taiwan; 6 Department of Anesthesiology, National Defense Medical Center, Taipei, Taiwan; 7 Department of Surgery, Chi Mei Medical Center, Tainan, Taiwan; 8 Department of Health and Nutrition, Chia Nan University of Pharmacy and Science, Tainan, Taiwan; 9 Department of Medical Research, Kaohsiung Medical University Hospital, Kaohsiung, Taiwan; 10 Center for Medical Informatics and Statistics, Office of R&D, Kaohsiung Medical University, Kaohsiung, Taiwan; 11 Center for Big Data Research, Kaohsiung Medical University, Kaohsiung City, Taiwan; 12 Department of Emergency Medicine, Kaohsiung Medical University, Kaohsiung, Taiwan; 13 Department of Healthcare Administration and Medical Informatics, Kaohsiung Medical University, 100 Shin-Chuan 1st Road, Kaohsiung, 807 Taiwan

**Keywords:** Cancer, Oncology

## Abstract

Most new cases and the highest mortality rates of breast cancer occur among middle-aged and old women. The recurrence rate of early-stage invasive ductal carcinoma (IDC) among women aged ≥ 50 years and receiving different treatments remains unclear. Therefore, this study was conducted to determine these rates. We used Surveillance, Epidemiology, and End Results (SEER) data for this nationwide population-based cohort study. All women aged ≥ 50 years and diagnosed with early-stage IDC between 2000 and 2015 were identified and divided into three treatment groups, namely, breast conservation therapy (BCT), mastectomy alone (MAS), and mastectomy with radiation therapy (MAS + RT). The recurrence rates of IDC among these groups were then compared. The BCT group had a lower short-term recurrence risk than the MAS and MAS + RT groups (hazard ratio [HR]: 1.00 vs. 2.90 [95% CI 1.36–2.66] vs. 2.07 [95% CI 0.97–4.44]); however, the BCT group also had a higher long-term recurrence risk than MAS and MAS + RT groups (HR 1.00 vs. 0.30 [95% CI 0.26–0.35] vs. 0.43 [95% CI 0.30–0.63]). The high long-term recurrence rate of the BCT group was especially prominent at the 10- and 15-year follow-ups. The results provide valuable evidence of the most reliable treatment strategy for this population. Further studies including more variables and validation in other countries are warranted to confirm our findings.

## Introduction

Breast cancer presents a great burden to public health and an important threat to women. According to breast cancer statistics in the United States, approximately 12% of all American women will develop invasive breast cancer in their lifetime^1^. Indeed, 276,480 new cases of female invasive breast cancer and 42,170 deaths from this disease are expected to occur in the country in 2020^1^. Second only to that of lung cancer, the mortality rate of breast cancer is higher than the mortality rates of other types of cancer^1^. In Taiwan, breast cancer is the most common female cancer, and this cancer was third leading cause of female cancer-related deaths in 2019^2^. The incidence rate of breast cancer is approximately 188–194 per 100,000 women^2^.

Breast cancer is more common in middle-aged and old women than in younger women, and most deaths are recorded in women aged ≥ 65 years^3^. In 2017, women aged > 50 years made up 81% of all new female cases of invasive breast cancer in the United States^4^. In Taiwan, 66.6% of the women diagnosed with breast cancer in 2017 were aged > 50 years^5^. Invasive ductal carcinoma (IDC) is the most common type of invasive breast cancer (80%); invasive lobular carcinoma (7%–15%) ranks a distant second in terms of invasiveness^6^. According to statistics in Taiwan, IDC comprises approximately 85.7% of all newly diagnosed breast cancer cases^5^. Therefore, the development of suitable treatment strategies, especially for early-stage IDC, is an important issue for the female population. Common treatments for early-stage IDC include (1) breast conservation therapy (BCT), which involves breast-conserving surgery (BCS) plus postsurgical radiation, (2) mastectomy alone (MAS), and (3) mastectomy with radiation therapy (MAS + RT)^7^.

A previous nationwide population-based study by the Surveillance, Epidemiology, and End Results (SEER) database revealed that women with early-stage IDC receiving BCT have better 5- and 10-year survival rates than those receiving MAS or MAS + RT^7^. However, this study included women aged ≥ 18 years, and the characteristics of this population may differ from those of middle-aged and old women. Few studies on recurrence rates following different treatments in middle-aged and old women with early-stage IDC have been published. Local recurrence is important to overall survival because local failure predicts distant metastasis in the future^8^. We conducted this nationwide population-based cohort study to assess the short- and long-term recurrence rates of early-stage IDC in middle-aged and old women following different treatments. Our hypothesis is that women receiving BCT will have a lower recurrence rate than those receiving MAS or MAS + RT.

## Materials and methods

### Data sources

We used SEER data reported by the National Cancer Institute for this study^9^. The SEER is a national population-based report of the most recent cancer incidence, prevalence, demographic characteristics, diagnosis time, tumor characteristics, surgery, RT, mortality, survival, and lifetime risk statistics in the United States^10^. It is published annually by the Surveillance Research Program of the National Cancer Institute in an effort to reduce the cancer burden among the United States population^10^. In the initial phase of the survey, seven registries (SEER 7) with epidemiologically significant population subgroups of racial and ethnic minorities were published. Since then, the database has been incrementally expanded to include 18 cancer registries (SEER 18)^11^. The SEER data can be applied for the analyses online.

### Study design, setting, and participants

**Figure 1:**
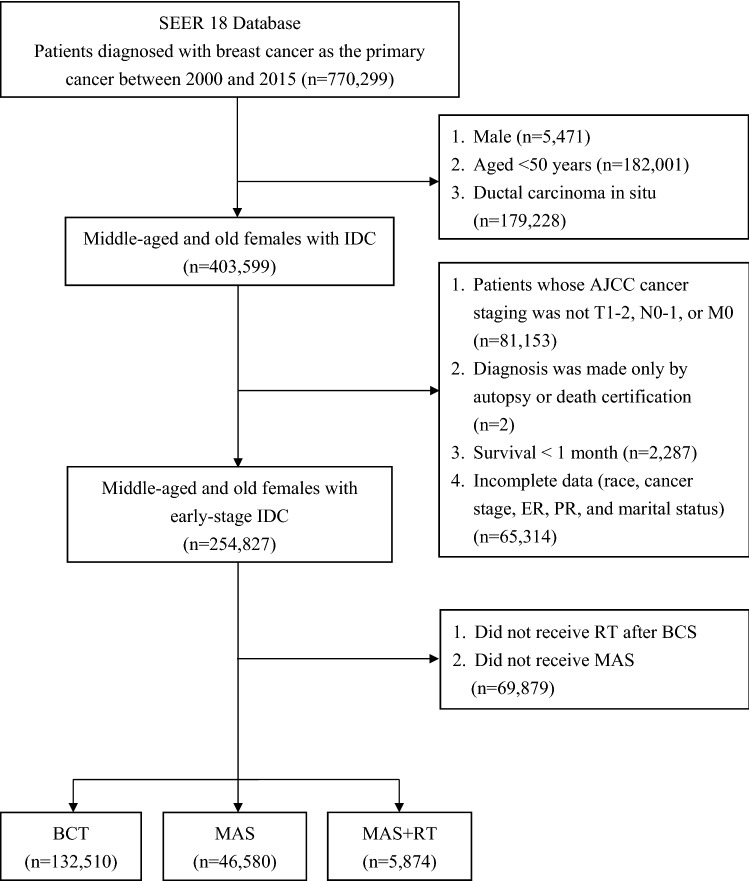
Flowchart of this study. SEER, Surveillance, Epidemiology, and End Results; IDC, invasive ductal carcinoma; AJCC, American Joint Committee on Cancer; ER, estrogen receptor; PR, progesterone receptor; RT, radiotherapy; BCS, breast conservative surgery; BCT, breast conservative treatment (BCS + RT); MAS, mastectomy alone.

We used the SEER 18 database to conduct a nationwide population-based cohort study. Initially, all patients diagnosed with breast cancer as the primary cancer between 2000 and 2015 were identified (Fig. 1). The exclusion criteria were as follows: (1) male; (2) aged < 50 years; (3) ductal carcinoma in situ; (4) American Joint Committee on Cancer (AJCC) cancer staging was not T1-2, N0-1, or M0; (5) diagnosis was made only by autopsy or death certification; (6) survival < 1 month; (7) incomplete data (race, cancer stage, estrogen receptor [ER], progesterone receptor [PR], and marital status); (8) did not receive RT after BCS and did not receive MAS. Finally, middle-aged, and old women (age ≥ 50 years) diagnosed with early-stage IDC as the primary cancer between 2000 and 2015 were identified for the analyses. According to the AJCC, the definitions of early-stage IDC are as follows: (1) cancer stage: T1-2, N0-1, or M0; (2) positive lymph nodes ≤ 3 (patients with > 4 positive lymph nodes were excluded because RT is almost suggested in these patients); (3) tumor size < 5 cm^12^. Patients were divided into three treatment groups as follows: (1) BCT (BCS + RT), (2) MAS, and (3) MAS + RT.

### Definitions of variables and outcomes

Age was divided into the following subgroups: (1) 50–59 years, (2) 60–69 years, (3) 70–79 years, (4) 80–89 years, and (5) ≥ 90 years (Table 1). Race was classified as white, black, and others. Marital status was classified as married, never married, widowed, and others. Tumor size was classified as ≤ 2 cm, 2–3 cm, 3–4 cm, and 4–5 cm. Tumor grade was classified as I, II, III, and IV based on histological findings. Positive lymph node(s) was classified as 0, 1, 2, and 3. ER and PR status were classified as positive and negative.

**Table 1:** Comparison of demographic and clinical characteristics among female patients with early-stage IDC receiving different treatments.

	Overall n = 184,964 100%	BCT n = 132,510 71.6%	MAS n = 46,580 25.2%	MAS + RT n = 5874 3.2%	*p*-value
Age	64.9 ± 9.5	64.0 ± 9.0	67.7 ± 10.5	63.4 ± 9.4	< 0.001
**Age subgroup**					< 0.001
50–59	62,403 (33.7)	47,651 (36.0)	12,389 (26.6)	2363 (40.2)	
60–69	64,780 (35.0)	48,859 (36.9)	13,954 (30.0)	1967 (33.5)	
70–79	42,638 (23.1)	28,456 (21.5)	13,032 (28.0)	1150 (19.6)	
80–89	14,424 (7.1)	7364 (5.6)	6681 (14.3)	379 (4.5)	
≥ 90	719 (0.4)	180 (0.1)	524 (1.1)	15 (0.3)	
**Race**					< 0.001
White	152,979 (82.7)	111,883 (84.4)	36,664 (78.7)	4432 (75.5)	
Black	15,951 (8.6)	10,801 (8.2)	4385 (9.4)	765 (13.0)	
Others	16,034 (8.7)	9826 (7.4)	5531 (11.9)	677 (11.5)	
**Marital Status**					< 0.001
Married	109,911 (59.4)	82,327 (62.1)	24,264 (52.1)	3320 (56.5)	
Never married	22,629 (12.2)	16,641 (12.6)	5184 (11.1)	804 (13.7)	
Widowed	32,113 (17.4)	19,368 (14.6)	11,735 (25.2)	1010 (17.2)	
Others	20,311 (11.0)	14,174 (10.7)	5397 (11.6)	740 (12.6)	
**Tumor size**					< 0.001
≤ 2 cm	138,102 (74.7)	107,296 (80.8)	28,560 (61.3)	2246 (38.2)	
2–3 cm	32,386 (17.5)	19,123 (14.4)	11,515 (24.7)	1748 (29.8)	
3–4 cm	10,439 (5.6)	4679 (3.5)	4685 (10.1)	1075 (18.3)	
4–5 cm	4037 (2.2)	1412 (1.1)	1820 (3.9)	805 (13.7)	
**Tumor grade**					< 0.001
I	45,683 (24.7)	36,498 (27.5)	8544 (18.3)	641 (10.9)	
II	81,341 (44.0)	58,677 (44.3)	20,332 (43.7)	2332 (39.7)	
III	56,782 (30.7)	36,658 (27.7)	17,281 (37.1)	2843 (48.4)	
IV	1158 (0.6)	677 (0.5)	423 (0.9)	58 (1.0)	
**Positive lymph node(s)**					< 0.001
0	145,221 (78.5)	109,718 (82.8)	33,462 (71.8)	2041 (34.8)	
1	25,385 (13.7)	15,748 (11.9)	7969 (17.1)	1668 (28.4)	
2	9594 (5.2)	4917 (3.7)	3496 (7.5)	1181 (20.1)	
3	4764 (2.6)	2127 (1.6)	1653 (3.6)	984 (16.8)	
**ER status**					< 0.001
Negative	32,963 (17.8)	20,793 (15.7)	10,642 (22.9)	1528 (26.0)	
Positive	152,001 (82.2)	111,717 (84.3)	35,938 (77.2)	4346 (74.0)	
**PR status**					< 0.001
Negative	53,561 (29.0)	34,961 (26.4)	16,321 (35.0)	2279 (38.8)	
Positive	131,403 (71.0)	97,549 (73.6)	30,259 (65.0)	3595 (61.2)	

Data are presented as n (%) or mean ± standard deviation. IDC, invasive ductal carcinoma; BCT, breast conservative treatment; MAS, mastectomy; RT, radiotherapy; ER, estrogen receptor; PR, progesterone receptor.

The primary outcomes were short-term recurrence rate (< 1 year) and long-term recurrence rate (≥ 1 year). Recurrence times were tracked beginning on the day breast cancer was first diagnosed. Recurrence was defined as local tumor recurrence in the breast (after BCT), chest wall (after MAS), ipsilateral/parasternal/infra- or supraclavicular lymph nodes, and skin of the chest wall (not breast)^13^. Because the time of surgical resection of tumors is not available in the database we used, we choose to use the time of diagnosis as the beginning of recurrence-free survival according to previous study using the same database^14^.

### Statistical analysis

For descriptive statistics, we used frequencies and percentages to represent categorical variables and means with standard deviations (SDs) to represent continuous variables. For inferential statistics, we used the chi-squared test to investigate associations between the three treatment groups and categorical variables in the demographic and clinical characteristics. One-way ANOVA (analysis of variance) was used to investigate associations between the three treatment groups and continuous variables in the demographic characteristics. Kaplan–Meier analysis and log-rank tests were used to compare differences in the recurrence curves of the three treatment groups. The Cox proportional hazard model was used to investigate predictors for recurrence. We used SAS 9.4 to obtain descriptive and inferential statistics and STATA SE13.0 to draw the recurrence curves. The significance level was set to 0.05 (two-tailed).

### Ethics approval and consent to participate

This study protocol was approved by the Institutional Review Board of Kaohsiung Medical University (Approval No. KMUHIRB-EXEMPT(II)-20190018). Informed consent was waived because we used deidentified secondary data from the SEER. The waiver does not affect the rights and welfare of the participants.

## Results

Overall, 184,964 patients were included in this study (Table 1). The BCT group included 132,510 patients (71.6%), the MAS group included 46,580 patients (25.2%), and the MAS + RT group included 5874 patients (3.2%). The mean age was 64.9 years, more patients were in the 60–69-year subgroup than in other subgroups, and patients in the MAS group tended to be older than those in other groups. Most patients were white (82.7%). Most of the patients were married (59.4%) or widowed (17.4%). The most common tumor size was ≤ 2 cm (74.7%), and most patients in the BCT group had tumors of this size (80.8%). In terms of tumor grade, grade II tumors were the most common (44.0%), followed by grade III tumors (30.7%). In terms of lymph node involvement, zero positive lymph nodes (78.5%) were the most common, especially in the BCT group (82.8%). The MAS + RT group had a higher percentage of three positive lymph nodes than the BCT and MAS groups. ER- and PR-positive tumors were present in 82.2% and 71.0%, respectively, of the total population and more common in the BCT group than in other groups.

**Figure 2:**
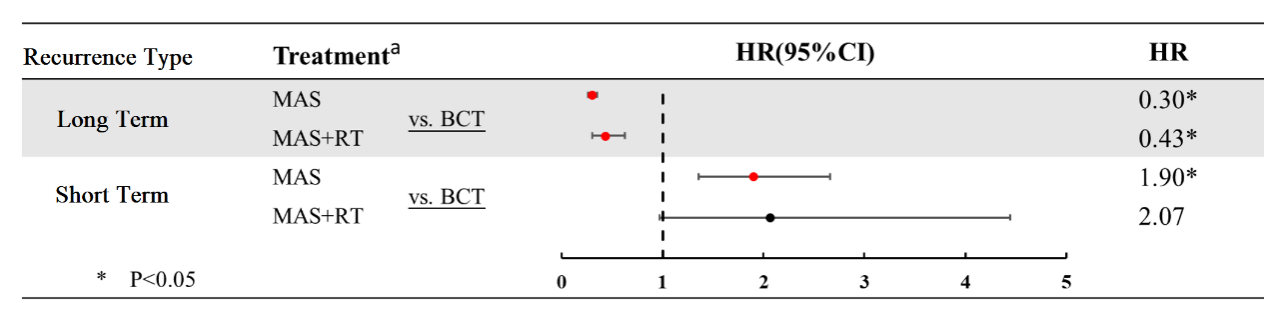
Comparison of short- and long-term recurrence rates among female patients with early-stage IDC receiving different treatments. IDC, invasive ductal carcinoma; HR, hazard ratio; CI, confidence interval; BCT, breast conservative treatment; MAS, mastectomy; RT, radiotherapy.

The BCT group had a lower short-term recurrence rate than the MAS and MAS + RT groups (0.07% vs. 0.14% vs. 0.14%; Table 2). Multivariate Cox regression analysis also showed that the BCT group has a lower short-term recurrence risk than the MAS and MAS + RT groups (hazard ratio [HR]: 1.00 vs. 2.90 [95% CI 1.36–2.66] vs. 2.07 [95% CI 0.97–4.44]; Table 3 and Fig. 2). By contrast, the BCT group had a higher long-term recurrence rate than the MAS and MAS + RT groups (1.2% vs. 0.4% vs. 0.5%) (Table 2). Multivariate Cox regression analysis showed that the BCT group has a higher long-term recurrence risk than the MAS and MAS + RT groups (HR 1.00 vs. 0.30 [95% CI 0.26–0.35] vs. 0.43 [95% CI 0.30–0.63]; Table 4 and Fig. 2). In addition, in the short-term recurrence analysis, only age 80–89 years was an independent predictor of recurrence (Table 3). Age ≥ 90 years, black race, tumor grade II, and PR-negative tumors reasonably predicted long-term recurrence (Table 4).

**Table 2:** Comparison of short-term and long-term recurrence rates among treatments and demographic characteristics in female patients with early-stage IDC.

	Short-term recurrence			Long-term recurrence		
	Recurrence	Non-recurrence	*p*-value	Recurrence	Non-recurrence	*p*-value
	n = 168	n = 184,796		n = 1822	n = 183,142	
**Treatment**			< 0.001			< 0.001
BCT	95 (0.07)	132,415 (99.93)		1619 (1.2)	130,891 (98.8)	
MAS	65 (0.14)	46,515 (99.86)		174 (0.4)	46,406 (99.6)	
MAS + RT	8 (0.14)	5866 (99.86)		29 (0.5)	5845 (99.5)	
**Age subgroup**			0.002			< 0.001
50–59	40 (0.1)	62,363 (99.9)		756 (1.2)	61,647 (98.8)	
60–69	61 (0.1)	64,719 (99.9)		624 (1.0)	64,156 (99.0)	
70–79	41 (0.1)	42,597 (99.9)		360 (0.8)	42,278 (99.2)	
80–89	24 (0.2)	14,400 (99.8)		75 (0.5)	14,349 (99.5)	
≥ 90	2 (0.3)	717 (99.7)		7 (1.0)	712 (99.0)	
**Race**			0.267			< 0.001
White	146 (0.1)	152,833 (99.9)		1533 (1.0)	151,446 (99.0)	
Black	13 (0.1)	15,938 (99.9)		187 (1.2)	15,764 (98.8)	
Others	9 (0.1)	16,025 (99.9)		102 (0.6)	15,932 (99.4)	
**Marital Status**			0.043			0.009
Married	92 (0.08)	109,818 (99.9)		1145 (1.04)	108,765 (98.96)	
Never married	20 (0.09)	22,609 (99.91)		224 (0.99)	22,405 (99.01)	
Widowed	37 (0.12)	32,076 (99.88)		276 (0.86)	31,837 (99.14)	
Others	19 (0.09)	20,292 (99.91)		177 (0.87)	20,134 (99.13)	
**Tumor size**			0.533			< 0.001
≤ 2 cm	126 (0.1)	137,976 (99.9)		1486 (1.1)	136,616 (98.9)	
2–3 cm	32 (0.1)	32,354 (99.9)		255 (0.8)	32,131 (99.2)	
3–4 cm	9 (0.1)	10,430 (99.9)		64 (0.6)	10,375 (99.4)	
4–5 cm	1 (0.02)	4036 (99.98)		17 (0.4)	4020 (99.6)	
**Tumor grade**			0.966			< 0.001
I	41 (0.1)	45,642 (99.9)		394 (0.9)	45,289 (99.1)	
II	77 (0.1)	81,264 (99.9)		850 (1.0)	80,491 (99.0)	
III	49 (0.1)	56,733 (99.9)		556 (1.0)	56 226 (99.0)	
IV	1 (0.1)	1157 (99.9)		22 (1.9)	1136 (98.1)	
**Positive lymph node(s)**			0.376			0.307
0	124 (0.1)	145,097 (99.9)		1459 (1.0)	143,762 (99.0)	
1	26 (0.1)	25,359 (99.9)		241 (1.0)	25,144 (99.1)	
2	11 (0.1)	9583 (99.9)		81 (0.8)	9513 (99.2)	
3	7 (0.2)	4757 (99.9)		41 (0.9)	4723 (99.1)	
**ER status**			0.07			< 0.001
Negative	21 (0.1)	32,942 (99.9)		406 (1.2)	32,557 (98.8)	
Positive	147 (0.1)	151,854 (99.9)		1416 (0.9)	150,585 (99.1)	
**PR status**			0.652			< 0.001
Negative	46 (0.1)	53,515 (99.9)		647 (1.2)	52,914 (98.8)	
Positive	122 (0.1)	131,281 (99.9)		1175 (0.9)	130,228 (99.1)	

Data are presented as n (%). IDC, invasive ductal carcinoma; BCT, breast conservative treatment; MAS, mastectomy; RT, radiotherapy; ER, estrogen receptor; PR, progesterone receptor.

**Table 3:** Comparison of short-term recurrence rates using univariate and multivariate analyses among treatment and demographic characteristics in patients with breast cancer.

Variable	Univariate analysis		Multivariate analysis	
	HR (95% CI)	*p*-value	HR (95% CI)	*p*-value
**Treatment**				
BCT	1.00 (reference)		1.00 (reference)	
MAS	1.95 (1.42–2.67)	< 0.001	1.90 (1.36–2.66)	< 0.001
MAS + RT	1.89 (0.92–3.90)	0.083	2.07 (0.97–4.44)	0.061
**Age group**				
50–59	1.00 (reference)		1.00 (reference)	
60–69	1.47 (0.99–2.20)	0.057	1.43 (0.96–2.14)	0.081
70–79	1.50 (0.97–2.32)	0.067	1.36 (0.87–2.15)	0.180
80–89	2.60 (1.57–4.32)	< 0.001	2.15 (1.24–3.75)	0.007
≥ 90	4.40 (1.06–18.19)	0.041	3.31 (0.77–14.25)	0.108
**Race**				
White	1.00 (reference)		1.00 (reference)	
Black	0.86 (0.49–1.51)	0.592	0.89 (0.50–1.59)	0.691
Others	0.59 (0.30–1.16)	0.126	0.58 (0.30–1.14)	0.113
**Marital status**				
Married	1.00 (reference)		1.00 (reference)	
Never married	1.06 (0.65–1.71)	0.823	1.05 (0.65–1.71)	0.843
Widowed	1.38 (0.94–2.02)	0.101	1.01 (0.66–1.54)	0.970
Others	1.12 (0.69–1.84)	0.648	1.13 (0.68–1.85)	0.643
**Tumor size**				
≤ 2 cm	1.00 (reference)		1.00 (reference)	
2–3 cm	1.09 (0.74–1.60)	0.680	0.91 (0.60–1.37)	0.653
3–4 cm	0.95 (0.48–1.86)	0.876	0.71 (0.35–1.44)	0.347
4–5 cm	0.27 (0.04–1.95)	0.196	0.19 (0.03–1.40)	0.103
**Tumor grade**				
I	1.00 (reference)		1.00 (reference)	
II	1.05 (0.72–1.54)	0.790	1.02 (0.70–1.50)	0.902
III	0.96 (0.63–1.45)	0.841	1.07 (0.67–1.69)	0.780
IV	0.95 (0.13–6.90)	0.958	1.02 (0.14–7.52)	0.982
**Positive lymph node(s)**				
0	1.00 (reference)		1.00 (reference)	
1	1.20 (0.79–1.83)	0.400	1.14 (0.74–1.75)	0.565
2	1.34 (0.72–2.49)	0.351	1.23 (0.65–2.32)	0.524
3	1.71 (0.80–3.67)	0.166	1.55 (0.70–3.43)	0.275
**ER status**				
Negative	1.00 (reference)		1.00 (reference)	
Positive	1.52 (0.97–2.41)	0.071	1.78 (1.00–3.18)	0.051
**PR status**				
Negative	1.00 (reference)		1.00 (reference)	
Positive	1.09 (0.77–1.52)	0.638	0.84 (0.55–1.28)	0.417

HR, hazard ratio; CI, confidence interval; BCT, breast conservative treatment; MAS, mastectomy; RT, radiotherapy; ER, estrogen receptor; PR, progesterone receptor.

**Table 4:** Comparison of long-term recurrence rates by using univariate and multivariate analyses among treatment and demographic characteristics in patients with breast cancer.

Variable	Univariate analysis		Multivariate analysis	
	HR (95% CI)	*p*-value	HR (95% CI)	*p*-value
**Treatment**				
BCT	1.00 (reference)		1.00 (reference)	
MAS	0.30 (0.26–0.35)	< 0.001	0.30 (0.26–0.35)	< 0.001
MAS + RT	0.43 (0.30–0.63)	< 0.001	0.43 (0.30–0.63)	< 0.001
**Age group**				
50–59	1.00 (reference)		1.00 (reference)	
60–69	0.93 (0.84–1.03)	0.178	0.96 (0.86–1.06)	0.400
70–79	0.85 (0.75–0.97)	0.012	0.93 (0.82–1.06)	0.285
80–89	0.69 (0.54–0.87)	0.002	0.85 (0.67–1.09)	0.209
≥ 90	2.27 (1.08–4.78)	0.031	3.42 (1.61–7.24)	< 0.001
**Race**				
White	1.00 (reference)		1.00 (reference)	
Black	1.44 (1.23–1.67)	< 0.001	1.42 (1.22–1.66)	< 0.001
Others	0.69 (0.56–0.84)	< 0.001	0.75 (0.62–0.92)	0.006
**Marital status**				
Married	1.00 (reference)		1.00 (reference)	
Never married	1.03 (0.89–1.19)	0.694	0.99 (0.85–1.14)	0.857
Widowed	0.93 (0.81–1.06)	0.258	1.04 (0.90–1.19)	0.634
Others	0.99 (0.85–1.16)	0.924	0.98 (0.84–1.15)	0.811
**Tumor size**				
≤ 2 cm	1.00 (reference)		1.00 (reference)	
2–3 cm	0.84 (0.73–0.96)	0.009	0.91 (0.79–1.04)	0.171
3–4 cm	0.72 (0.56–0.92)	0.009	0.85 (0.66–1.10)	0.217
4–5 cm	0.53 (0.33–0.85)	0.009	0.68 (0.42–1.10)	0.116
**Tumor grade**				
I	1.00 (reference)		1.00 (reference)	
II	1.20 (1.06–1.35)	0.003	1.24 (1.10–1.39)	< 0.001
III	1.16 (1.02–1.32)	0.025	1.12 (0.96–1.29)	0.142
IV	1.45 (0.94–2.22)	0.092	1.46 (0.95–2.26)	0.088
**Positive lymph node(s)**				
0	1.00 (reference)		1.00 (reference)	
1	0.94 (0.82–1.08)	0.383	1.04 (0.91–1.19)	0.584
2	0.84 (0.67–1.05)	0.127	1.00 (0.80–1.26)	0.998
3	0.85 (0.63–1.16)	0.314	1.08 (0.79–1.48)	0.633
**ER status**				
Negative	1.00 (reference)		1.00 (reference)	
Positive	0.79 (0.71–0.88)	< 0.001	0.88 (0.75–1.03)	0.118
**PR status**				
Negative	1.00 (reference)		1.00 (reference)	
Positive	0.78 (0.71–0.86)	< 0.001	0.80 (0.70–0.91)	< 0.001

HR, hazard ratio; CI, confidence interval; BCT, breast conservative treatment; MAS, mastectomy; RT, radiotherapy; ER, estrogen receptor; PR, progesterone receptor.

Subgroup analysis of short-term recurrence showed that the BCT group has a lower recurrence rate at 6 months (Supplementary Table 1 and Supplementary Fig. 1) than the other groups. Long-term recurrence analysis showed that the BCT group has a higher recurrence rate than the MAS and MAS + RT groups at the 10- and 15-year follow-ups (Supplementary Table 2 and Supplementary Fig. 2). The follow-up rates and numbers of subjects at risk at each follow-up time-points was showed in the Supplementary Table 3. The 1-yr, 3-yr, 5-yr cumulative recurrence rates and 95% CIs of three treatment groups was showed in the Supplementary Table 4. Competing risk analysis with adjustment for demographic and clinical characteristics revealed that the BCT group (reference) has a lower short-term recurrence risk than the MAS (HR 1.90, *p* < 0.001) and MAS + RT (HR 2.08, *p* = 0.048) groups. Moreover, the BCT group (reference) had a higher long-term recurrence risk than the MAS (HR 0.28, *p* < 0.001) and MAS + RT (HR 0.42, *p* < 0.001) groups. The proportionality assumption of Cox proportional hazard model was checked by plotting log minus log (log(−log(S(t))) vs. t) in the model. The parallelism in the plot indicates the proportionality assumption was satisfied (Supplementary Fig. 3).

## Discussion

The present study showed that most breast cancer patients receive BCT, followed by MAS and MAS + RT. Patients who received BCT were more likely to be of white race and have a smaller tumor size, lower tumor grade, fewer positive lymph nodes, and larger number of ER- and PR-positive tumors than patients in other groups. Compared with the MAS and MAS + RT groups, the BCT group had a lower short-term recurrence risk but a higher long-term recurrence risk, especially at the 10 and 15-year follow-ups. Age ≥ 90 years, black race, tumor grade II, and PR-negative tumors were independent predictors for long-term recurrence.

We confirmed that the BCT group has a higher long-term recurrence risk than the MAS and MAS + RT groups; this result sheds some light on what a long-disputed issue in the literature has been. The possible explanations for the higher long-term recurrence risk in the BCT group are incomplete surgical removal of tumor cells of precancerous lesions, subclinical lesions, or malignant cells not eradicated by RT^15,16^. A previous study using the same database with this study showed that the BCT group has a higher long-term survival rate than MAS group and MAS + RT group^7^. However, the present study focused on middle-aged and old women, different from the study recruiting all women ≥ 18 years^7^. Another difference is that the present study was conducted to investigate recurrence, not survival. Therefore, using BCT for prevention of recurrence rate in middle-aged and old women with early-stage IDC should be more cautious. Further investigation about the different effect is warranted.

Risk of recurrence has a great influence on patients with breast cancer because this risk causes patients to live with a constant fear of death^17^. The reasons behind local recurrence remain largely unknown^17^, but the possible mechanisms include the existence of cancer stem cells and transformation of cancer cells into a relatively aggressive phenotype^17^. Cancer stem cells and transformed cancer cells are highly metastatic and resistant to conventional therapies^17^. A high percentage of aggressive cells is a feature of recurrent breast cancers^17^. Many clinical predictors for recurrence, including ER-negative, PR-negative, human epidermal growth factor receptor 2 (HER-2)-positive, triple-negative breast cancers, age, race, menopausal status, smoking, mammographic features, tumor morphology, tumor size, tumor stage, lymph node metastases, and gene expression profiling, have been proposed^17,18^.

Age ≥ 90 years, which has not been fully studied in the literature, was identified to be an independent predictor for long-term recurrence in the present study. A large population-based study in the Netherlands in 2020 reported that patients aged 75–79 years were at higher risk of distant recurrence than patients aged 70–74 years (subdistribution HR 1.25; 95% CI 1.11–1.41); however, age ≥ 80 years did not show this higher risk^19^. The authors attributed their findings to several reasons: (1) patients in the aged 75–79 years were undertreated, (2) the risk of death without recurrence increases with age, and (3) patients with a high competing mortality risk were overtreated^19^. Another population-based study in Germany in 2019 revealed that patients aged < 70 years have higher 5- and 10-year locoregional recurrence and distant metastasis rates than those aged ≥ 70 years (17% vs. 13%)^20^. More evidence is needed to clarify this finding. Black race was a risk factor for cancer recurrence in the present study, consistent with findings in previous studies^18^. Racial disparities may be due to socioeconomic factors and a more aggressive tumor biology among African–Americans^18^. Tumor grade was also associated with poor outcomes^18^. The present study revealed that tumor grade II is associated with long-term recurrence. While patients with tumor grades III and IV were at higher risk for long-term recurrence than those with tumor grade I, the difference between grades was not significant. PR-negative is a predictor for recurrence, and the results between the present and previous studies are consistent^18^. In general, breast cancers that are single hormone receptor-positive appear to have a poorer prognosis than those that are both ER- and PR-positive^18^. The present study also revealed a higher long-term recurrence risk in patients with ER-negative breast cancer than in those with ER-positive breast cancer; however, the difference was not significant (HR 1.13; 95% CI 0.97–1.33).

The major strengths of the present study include its nationwide population-based design, large sample size, and clear delineation of the knowledge gap in research on the recurrence rate of early-stage IDC in women aged ≥ 50 years. The limitations are as follows. First, the data were obtained from various institutions and may have bias in terms of treatment and quality. Second, because the present study conducts a secondary analysis of data, the results can only suggest associations between variables rather than causal relationships. Third, some variables, including genetic data, lymphovascular invasion, size of metastatic lymph nodes, resection margins, adjuvant therapies (e.g., chemotherapy and endocrine therapy), and HER2, were not considered in the present study because data on these variables were made available only after 2010. Fourth, because the data used for our analyses are from the United States, their generalization to other countries requires further validation. In the future, we plan to use the breast cancer database in Taiwan to validate the finding in this study.

## Conclusion

This nationwide population-based cohort study revealed that, among middle-aged and old women with early-stage IDC, the BCT group has a lower short-term recurrence risk but a higher long-term recurrence risk than the MAS and MAS + RT groups, especially at the 10- and 15-year follow-ups. Using BCT should be cautious for its higher long-term recurrence in middle-aged and old women with early-stage IDC. The results fill the knowledge gap in research on the long- and short-term recurrence rates of IDC and provide valuable evidence of the most reliable treatment strategy for this population. Further studies, including more variables and validation in other countries, are warranted to confirm our findings.

## Supplementary Information

Supplementary Information.

## Data Availability

The data of SEER are publicly available. Please see the website https://seer.cancer.gov/archive/csr/1975_2014/.

## Abbreviations

IDC: Invasive ductal carcinoma
BCT: Breast conservation therapy
BCS: Breast-conserving surgery
MAS: Mastectomy
RT: Radiation therapy
SEER: Surveillance, epidemiology, and end results
AJCC: American Joint Committee on Cancer
ER: Estrogen receptor
PR: Progesterone receptor
SD: Standard deviation
ANOVA: Analysis of variance
HR: Hazard ratio
CI: Confidence interval
HER-2: Human epidermal growth factor receptor 2
