# Supplementary material for: Short- and long-term recurrence of early-stage invasive ductal carcinoma in middle-aged and old women with different treatments
Source: Sci Rep. 2022 Mar 15;12:4422. doi: 10.1038/s41598-022-08328-4 (PMC8924278; doi:10.1038/s41598-022-08328-4)
Supplement: Supplementary file 1 — Supplementary Information. [file 41598_2022_8328_MOESM1_ESM.docx]

**Supplementary Table 1.** Comparison of short-term recurrence rates (<6 and <12 months) among female patients with early-stage IDC receiving different treatments

| Treatment | <6 months | <12 months | *p*-value |
| --- | --- | --- | --- |
| BCT | 0.05% | 0.07% | <0.001 |
| MAS | 0.12% | 0.14% |  |
| MAS+RT | 0.10% | 0.14% |  |

IDC, invasive ductal carcinoma; BCT, breast conservative treatment; MAS, mastectomy; RT, radiotherapy.

**Supplementary Table 2.** Comparison of long-term recurrence rates among female patients with early-stage IDC receiving different treatments

| Treatment | <1 year | <3 years | <5 years | <10 years | <15 years | *p*-value |
| --- | --- | --- | --- | --- | --- | --- |
| BCT | 0.07% | 0.12% | 0.43% | 2.01% | 4.15% | <0.001 |
| MAS | 0.14% | 0.20% | 0.24% | 0.55% | 0.81% |  |
| MAS+RT | 0.14% | 0.24% | 0.43% | 0.68% | 0.97% |  |

IDC, invasive ductal carcinoma; BCT, breast conservative treatment; MAS, mastectomy; RT, radiotherapy.

**Supplementary Table 3.** Follow-up rates and numbers of subjects at risk at each follow-up time-points

| Treatment | N | Number at risk (follow-up rates) | | | | | |
| --- | --- | --- | --- | --- | --- | --- | --- |
|  |  | <6 months | <1 year | <3 years | <5 years | <10 years | <15 years |
| BCT | 132,510 | 127,435 | 121,220 | 97,930 | 75,976 | 31,832 | 4,257 |
|  |  | (96.2%) | (91.5%) | (73.9%) | (57.3%) | (24.0%) | (3.2%) |
| MAS | 46,580 | 44,863 | 42,975 | 35,029 | 27,248 | 11,355 | 1,413 |
|  |  | (96.3%) | (92.3%) | (75.2%) | (58.5%) | (24.4%) | (3.0%) |
| MAS+RT | 5,874 | 5,693 | 5,425 | 4,303 | 3,196 | 1,237 | 187 |
|  |  | (96.9%) | (92.4%) | (73.3%) | (54.4%) | (21.1%) | (3.2%) |

BCT, breast conservative treatment; MAS, mastectomy; RT, radiotherapy.

**Supplementary Table 4.** The 1-yr, 3-yr, 5-yr cumulative recurrence rates and 95% confidence intervals of the three treatment groups

| Treatment | <1 year | <3 year | <5 year |
| --- | --- | --- | --- |
| BCT | 0.07% (0.06%−0.09%) | 0.20% (0.18%−0.23%) | 0.43% (0.39%−0.47%) |
| MAS | 0.14% (0.11%−0.18%) | 0.20% (0.16%−0.25%) | 0.24% (0.20%−0.29%) |
| MAS+RT | 0.14% (0.07%−0.28%) | 0.24% (0.14%−0.41%) | 0.43% (0.27%−0.67%) |

BCT, breast conservative treatment; MAS, mastectomy; RT, radiotherapy

**Supplementary Figure 1.** Comparison of short-term recurrence rates among female patients with invasive ductal carcinoma receiving different treatments. BCT, breast conservative treatment; MAS, mastectomy; RT, radiotherapy


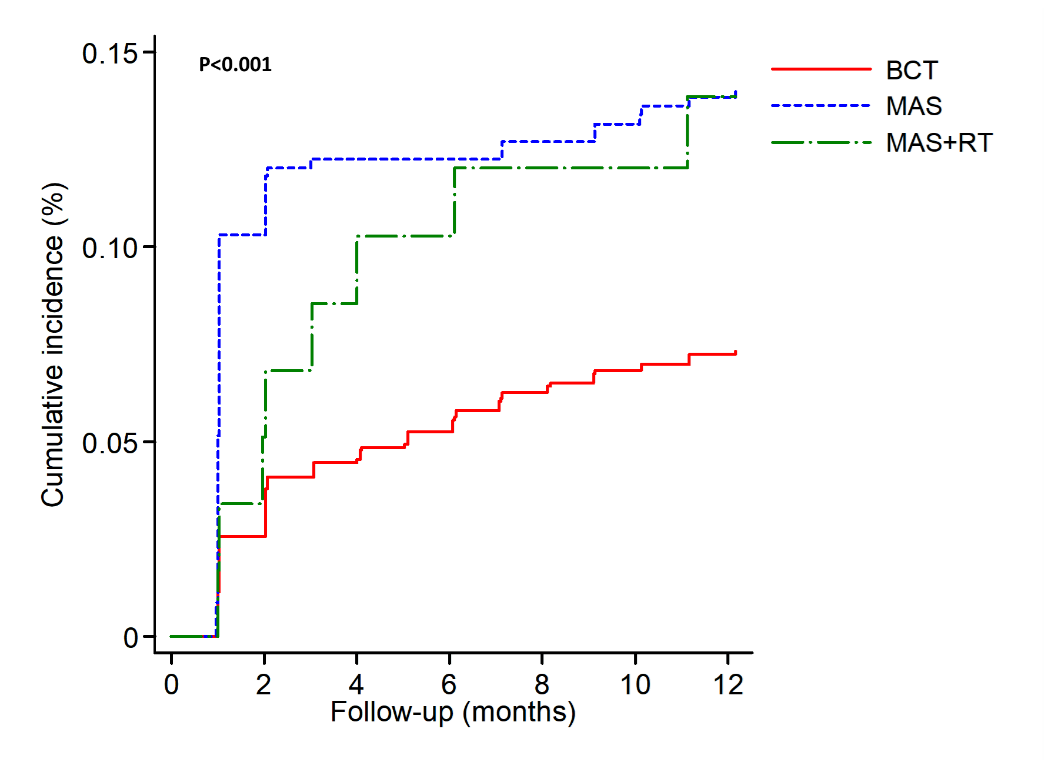


**Supplementary Figure 2.** Comparison of long-term recurrence rates among female patients with invasive ductal carcinoma receiving different treatments. BCT, breast conservative treatment; MAS, mastectomy; RT, radiotherapy


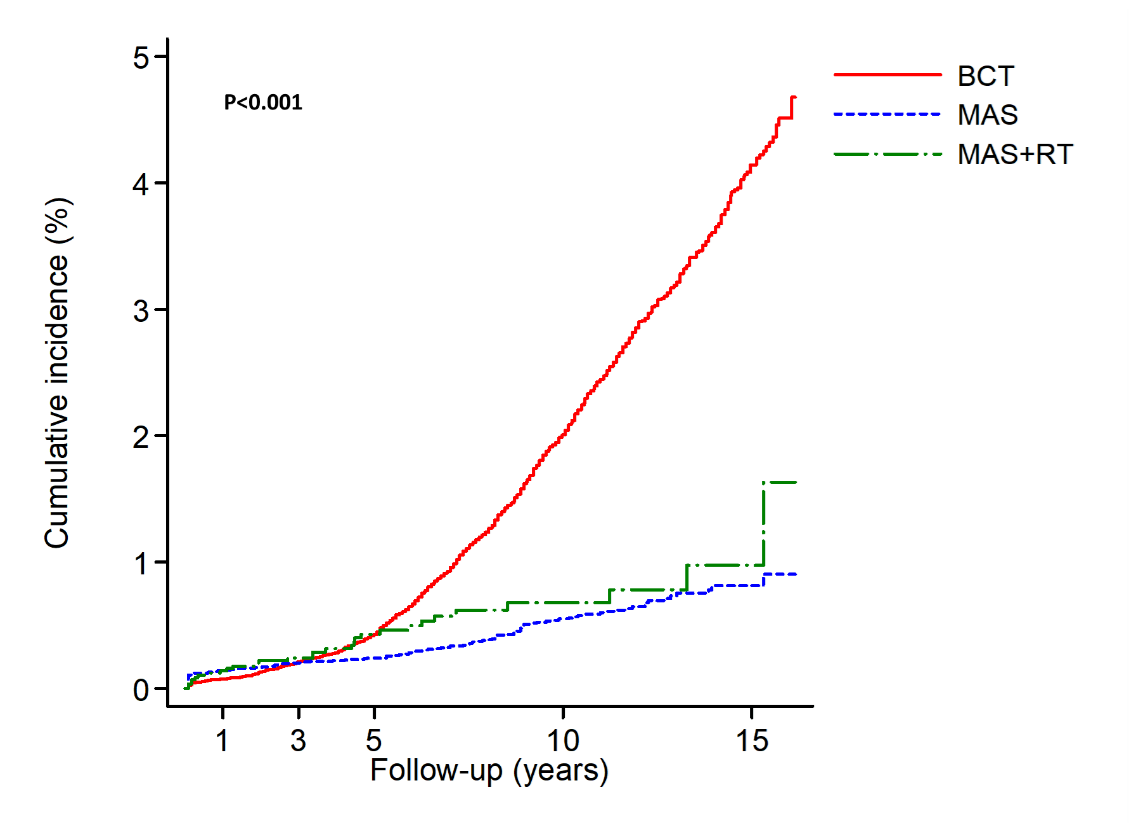


**Supplementary Figure 3.** Log minus Log Plot for checking the proportionality assumption of Cox proportional hazard model

| (1) short-term recurrence | (2) long-term recurrence |
| --- | --- |
| 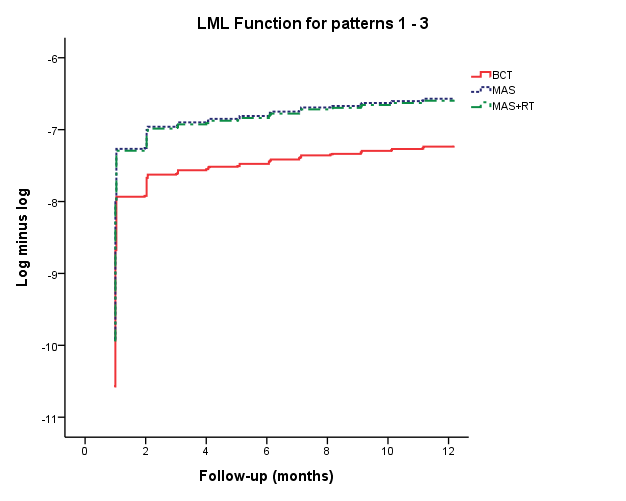 | 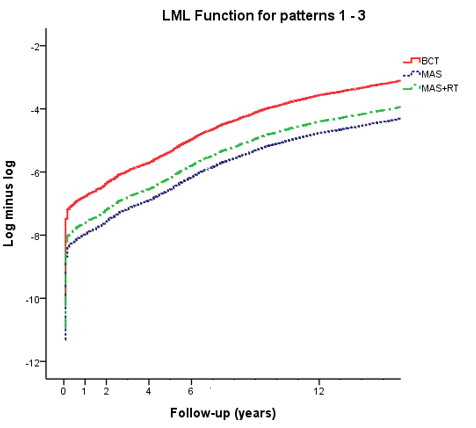 |
